# For ultra-high dose rate carbon-ion irradiation, comparable beam parameters induce the equivalent cell sparing (FLASH) effect

**DOI:** 10.1093/jrr/rrag039

**Published:** 2026-06-03

**Authors:** Kento Tsubouchi, Yukari Yoshida, Masashi Yagi, Kazumasa Minami, Hiromu Suda, Masao Nakao, Ken Yusa, Mutsumi Tashiro, Shinichi Shimizu, Tatsuya Ohno, Kazuhiko Ogawa

**Affiliations:** Department of Carbon Ion Radiotherapy, The University of Osaka Graduate School of Medicine, 2‑2 Yamada‑oka, Suita, Osaka 565-0871, Japan; Gunma University Heavy Ion Medical Center, 3-39-22 Showa-machi, Maebashi, Gunma 371-8511, Japan; Division of Radiation Oncology, National Cancer Centre Singapore, 30 Hospital Boulevard, Singapore 168583, Singapore; Department of Radiation Oncology, The University of Osaka Graduate School of Medicine, 2‑2 Yamada‑oka, Suita, Osaka 565-0871, Japan; Division of Health Science, The University of Osaka Graduate School of Medicine, 1-7 Yamada-oka, Suita, Osaka 565-0871, Japan; Gunma University Heavy Ion Medical Center, 3-39-22 Showa-machi, Maebashi, Gunma 371-8511, Japan; Gunma University Heavy Ion Medical Center, 3-39-22 Showa-machi, Maebashi, Gunma 371-8511, Japan; Gunma University Heavy Ion Medical Center, 3-39-22 Showa-machi, Maebashi, Gunma 371-8511, Japan; Gunma University Heavy Ion Medical Center, 3-39-22 Showa-machi, Maebashi, Gunma 371-8511, Japan; Department of Carbon Ion Radiotherapy, The University of Osaka Graduate School of Medicine, 2‑2 Yamada‑oka, Suita, Osaka 565-0871, Japan; Gunma University Heavy Ion Medical Center, 3-39-22 Showa-machi, Maebashi, Gunma 371-8511, Japan; Department of Radiation Oncology, Gunma University Graduate School of Medicine, 3-39-22 Showa-machi, Maebashi, Gunma 371-8511, Japan; Department of Radiation Oncology, The University of Osaka Graduate School of Medicine, 2‑2 Yamada‑oka, Suita, Osaka 565-0871, Japan

**Keywords:** flash effect, carbon ion beam, cell sparing effect, different machines, different facility

## Abstract

Recently, ultra-high dose rate (uHDR) irradiation has received attention for FLASH effect, a phenomenon *in vivo* that reduces normal-tissue damage without compromising antitumor efficacy compared to normal dose rate (NDR) irradiation. Such protective responses observed *in vitro* are referred to as cell-sparing effect. In previous studies, the cell-sparing effect was demonstrated using a carbon-ion beam scanning system. This study aimed to reproduce comparable irradiation conditions using a different machine at another facility and evaluate the cell-sparing effect. Comparable beam settings were adopted: physical dose 7 Gy, average dose rates (ADR) 100 Gy/s for uHDR and 1 Gy/s for NDR, dose-averaged linear energy transfer (LETd) 16.3 and 50 keV/μm, and identical scanning patterns. The same cell lines, Human salivary gland cell line (HSGc-C5), human dermal fibroblast (HDF), and human lung bronchial epithelial cell line (Nuli-1), were irradiated under normoxia. Colony formation assay and immunofluorescence staining of γH2AX were performed to assess cell survival and DNA damage. Comparable physical dose, ADR, and field flatness were verified by measurements. HDF and Nuli-1 showed the cell-sparing effects with increased surviving fractions and less DNA damage, which were enhanced at higher LET. In contrast, HSGc-C5 exhibited the smaller cell-sparing effect, being absent at low LET. These results were largely consistent with previous studies. To the best of current knowledge, this study is the first to indicate that the cell-sparing effect depends not on irradiation devices but on beam parameters, contributing to accelerating further FLASH research and clinical implementation of carbon-ion uHDR irradiation.

## INTRODUCTION

Recently, ionizing irradiation at ultra-high dose rate (uHDR) has attracted increasing attention due to the effect of protecting normal tissues without attenuating the damage to target tumors compared with normal dose rate (NDR) irradiation *in vivo*, so-called ‘FLASH effect.’ Since Favaudon *et al.* revealed this phenomenon, mitigating damage to normal lungs caused by uHDR (> 40 Gy/s) electron irradiation [1], there have been many experiments *in vivo* to assess the FLASH effect of several tissues, such as skins [2], muscles [3], lungs [1, 4], brain [5, 6], and abdominal tissues [4, 7, 8], immune systems [7], and many types of tumors [1, 3, 8, 9] in murine model [1–9] and zebrafish embryo [5, 10, 11]. In addition, *in vitro* studies revealed uHDR irradiation reduced damage to normal cells [12–16] and in some cases tumor cells [5, 15–17]. Such protective effects on both tumor and normal tissues and cells, observed under specific conditions in both *in vivo* and *in vitro* studies, are collectively referred to as ‘sparing effect,’ which is distinct from the FLASH effect observed only *in vivo* [18]. In *in vitro* studies, this phenomenon is termed ‘cell-sparing effect.’ Investigation into the FLASH and cell-sparing effect was performed using electron [1, 2, 5, 7, 8, 10, 12, 13, 17], including very high energy electron (VHEE), proton [15, 19–22], photon [4, 6], carbon-ion [3, 9, 14, 16, 23, 24] and the other heavy-ion beams [11]. Notably for electron and proton beam, clinical trial using uHDR irradiation has been performed [25–27]. However, underlying mechanisms of the FLASH and cell-sparing effect have not been fully understood though some hypothesis such as oxygen depletion, recombination of radicals, immune response and mitochondrial responses have been explored with *in vivo*, *in vitr*o and *in silico* studies [2, 12, 13, 18, 28–32].

The required conditions to induce the FLASH and cell-sparing effect have been assessed in many articles. From the perspective of physical parameters, basic beam parameters including not only dose rate, but beam energy, physical dose, irradiation time, and field size have been investigated in several types of ionizing beams. In addition, types of ion accelerator have several variations such as linear accelerator, cyclotron, synchrocyclotron, and synchrotron. Furthermore, for particle beams, irradiation techniques depend on irradiation systems, such as the double-scattering method and the scanning methods. Moreover, dose averaged linear energy transfer (LETd) value, which reflects radical density along particle tracks and the radical recombination hypothesis [33], are also important to assess the biological responses and is used in some biological dose calculation models of particle beam [9, 34–42]. Additionally, in the literature of the uHDR irradiation of pulsed beam, the time structures such as pulse width, pulse repetition frequency (PRF), dose and dose rate in a single pulse (DPP and $ \dot{\mathrm D}$_p_), have been considered non-negligible [7, 8, 10, 28, 29].

In terms of biological parameters, various types of biomaterials, such as types of animals, tissues, cell lines, and experimental approaches with various endpoints to assess the damages induced by the irradiation were utilized. On the basis of some hypothesis, such as oxygen depletion and radical recombination hypothesis, oxygen concentrations has a key role in the physiochemical and chemical stage of radiation reaction [2, 12–14, 16, 18, 23, 29–32]. Also immune systems have been regarded as important in tumor response [18, 24].

As mentioned above, the required beam parameters and underlying mechanism for the FLASH and cell-sparing effect have been assessed based on each experimental method using each irradiation setting and each biological material. Such methodological variations make it difficult to interpret and generalize the results. For uHDR irradiation, specialized machine or some dedicated modifications to the clinical machine setting were required to deliver and control extremely dense carbon-ion particles, such as the introduction of a dedicated monitor chamber and the modification of beam extraction methods [23, 43, 44]. Thus, inter-facility comparison dedicated to uHDR carbon-ion irradiation dosimetry and biological effects is needed to verify the required condition for FLASH and cell-sparing effect. To the best of current knowledge, there have been a few reports of inter-facility comparison to assess whether the same beam settings using another machine induce the comparable FLASH and cell-sparing effect [45, 46]. Especially for carbon ion uHDR irradiation, there is no report as far as we know. There are only few reports of the FLASH and cell-sparing effect for carbon-ion beam based on *in vivo* [3, 9] and *in vitro* [14, 16, 23, 24] experiments because irradiation machines which can achieve uHDR irradiation are limited [34]. Therefore, it is important to confirm that the same beam parameters with appropriate verification can induce the comparable sparing effect for driving the FLASH research to discover the underlying mechanism for uHDR carbon-ion beam forward and the clinical implementation in the future. We consider that simple experimental systems, such as cellular experiments, are suitable to compare the response to uHDR irradiation and uncover the underlying mechanism, and we conducted *in vitro* experiments in this study.

In previous research conducted at Osaka Heavy Ion Therapy Center (Osaka-HIMAK), uHDR irradiation conditions were established, and several requirements for cell-sparing effect induced by uHDR carbon-ion irradiation were identified [14, 32, 33]. In these studies, synchrotron-based machine (HyBEAT, Hitachi Ltd., Tokyo, Japan) and raster scanning method were adapted to uHDR carbon-ion irradiation to normal and tumor cell lines. For uHDR carbon-ion irradiation, basic beam parameters were set as follows; averaged dose rate (ADR) of 100 Gy/s, physical dose of 6.5 Gy and field size of approximately 20 × 20 mm^2^ composed of 7 × 7 spots under plateau and near Bragg-peak region, with LETd value of 19 and 50 keV/μm respectively. These irradiation conditions induced cell-sparing effects under both normoxia and hypoxia for normal cell lines and under only hypoxia for the tumor cell line. In addition, higher LET and lower oxygen concentration enhance sparing effect. This is the only report of the cell-sparing effect under normoxia for carbon-ion uHDR irradiation [16]. However, it is unclear whether these irradiation conditions generally meet the requirements for the cell-sparing effect. There is an inconsistent report showing that cell-sparing effect could not be observed after 7.5 Gy carbon-ion irradiation under normoxia, though there were differences in cell lines and experimental methods in detail [14]. The inter-facility comparison conducting the same biological experiments with same cell line is desired to verify these requirements.

This study first aimed to reproduce comparable beam parameters and irradiation conditions of the previous research [16, 43] including dose, dose rate, LETd, scanning method and oxygen concentration using a different machine at another facility [47]. Under these reproduced conditions, we evaluated the cell-sparing effect by conducting the same biological experiments.

## MATERIALS AND METHODS

### Carbon-ion irradiation system design

**Fig. 1:**
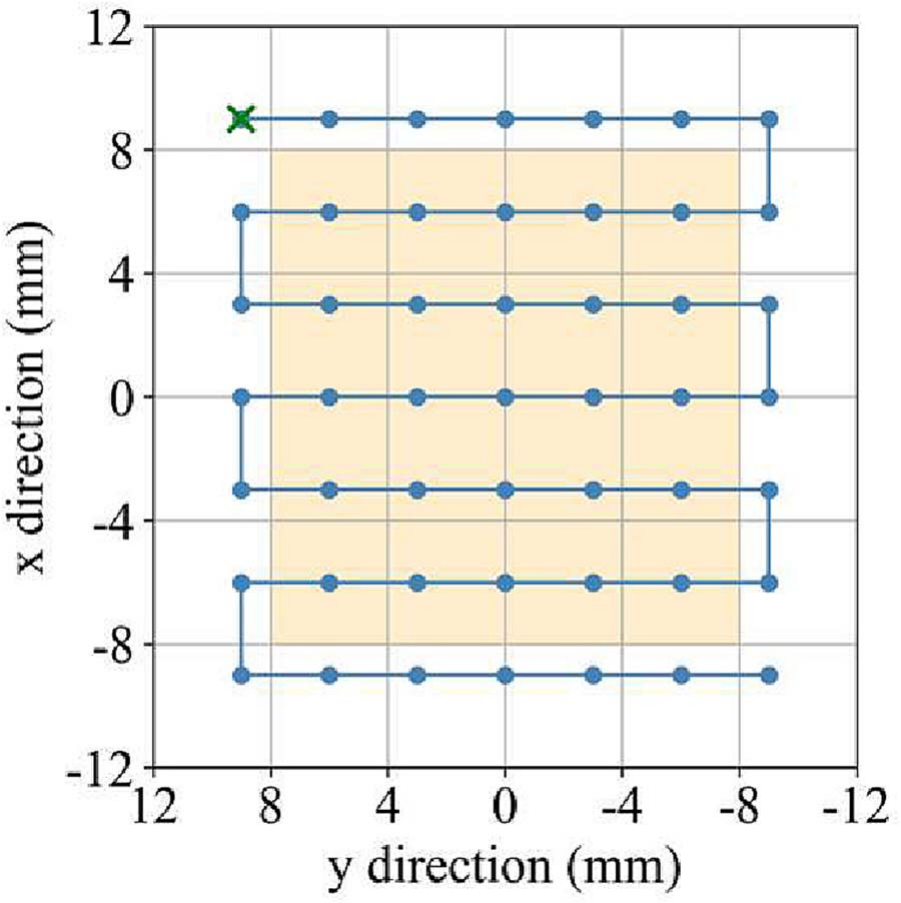
Scanning pattern used in this study. The green cross mark indicates the starting spot. The orange square area at the center of the figure, extending ±8 mm in both x- and y-directions, sufficient to cover the single well of 24-well plate, was used to analyze field flatness.

A vertical scanning port of synchrotron-based carbon-ion irradiation system (MELTHEA, Hitachi Ltd., Tokyo, Japan) at Gunma University Heavy Ion Medical Center (GHMC) [23, 47] were adopted to carbon-ion irradiation for cell experiments. Except for using raster scanning method and modification of some parameters, the irradiation was performed as described in the previous report [23]. Nominal beam energy was set to 290 MeV/u, with LETd value of 16.3 and 50.0 keV/μm under plateau region and near Bragg-peak region, respectively. The water-equivalent depth for each LETd setting was carefully adjusted by using a variable-water-thickness phantom and acrylic plates. Raster scanning method consisting of 7 × 7 spots with 3 mm spacing was used to make approximately 20 × 20 mm^2^ flat irradiation field large enough to conduct cell experiments. To simplify the time structure, irradiation was performed within single scan without repainting. Scanning patterns are shown in Fig. 1. The standard deviation (SD) of single spot profile was approximately 2.4 mm. In this study, two dose rate conditions, uHDR and NDR conditions were applied. ADR, calculated by total physical dose divided by total irradiation time, was set to 100 Gy/s for uHDR and 1 Gy/s for NDR conditions, respectively. For uHDR condition, irradiation was achieved within single spill.

### Validation of carbon-ion beam dosimetry

Physical dose was measured by Advanced Markus Chamber (PTW34045, PTW, Freiburg, Germany) connected to electrometer (KEITHLEY 6517A, Keithley Instruments LLC, Solon, Ohio, United States) with a bias voltage of 300 V, which is higher enough to neglect the recombination under even uHDR conditions [23, 43, 48]. The Advanced Markus Chamber was appropriately cross calibrated against a reference chamber with ensured traceability to national standard. The total irradiation time was assessed by the log data from monitor ion chamber. ADR was calculated based on the measured physical dose and assessed total irradiation time data. For the validation of dose distribution, Gafchromic films (EBT4, Ashland Inc., Wilmington, Delaware, USA) were placed at isocenter according to room lasers and irradiated under plateau and near Bragg-peak region. The irradiated films were scanned by flatbed scanner (EPSON ES-10000G, Seiko Epson Corporation, Nagano, Japan) at 96 dpi. Lateral dose profiles for x- and y-axis were extracted using ImageJ software [49] (version 1.54 g, U.S. National Institute of Health, Bethesda, Maryland, USA). Beam flatness in the range of ±8 mm in both x- and y-directions large enough to cover the single well of 24-well plate was calculated by following equation using maximum dose (D_Max_) and minimum dose (D_min_); (D_Max_ – D_min_) / (D_Max_ + D_min_).

### Cell lines

We employed the same tumor and normal cell lines as those used in the previous report [16]. The Human salivary gland cell line (HSGc-C5) was acquired from RIKEN (Tsukuba, Ibaraki, Japan) and Human dermal fibroblast (HDF) and Human lung bronchial epithelial cell line (Nuli-1) were obtained from ATCC (Manassas, VA, USA). These cell lines were cultured in Dulbecco’s Modified Eagle Medium (DMEM) (Thermo Fisher Scientific, Waltham, MA, USA) containing 10% fetal bovine serum (Thermo Fisher Scientific), 1% penicillin, streptomycin, and L-glutamine (NACALAI TESQUE, INC., Kyoto, Japan) at 37°C in a humidified 5% CO_2_ atmosphere.

### Cell irradiation

Cells were irradiated under normoxic conditions following the procedure reported previously [14]. The cells were cultured in 24-well plates and placed at isocenter according to room lasers and depth of plateau or near Bragg-peak region. The cells were irradiated with 7.0 Gy under uHDR or NDR condition. The physical dose 7.0 Gy, a little bit higher than the previous report was selected considering the possible uncertainty in physical dose.

### Colony formation assay

Immediately after irradiation, irradiated cells were washed in Phosphate Buffered Saline (PBS) and trypsinized. The cells were seeded to a 60 mm diameter dishes, cultured for 2 or 3 weeks, fixed with formalin, and stained with 0.25% crystal violet. The colonies comprising >50 cells were scored as survivors, and the surviving fraction was calculated. More than six independent samples for each condition were used for statistical analysis.

### γH2AX immunofluorescent staining

One hour after irradiation, cells were fixed using 4% Paraformaldehyde (Sigma Chemicals, Kewdale, WA, USA) for 30 min, and permeabilized with 0.5% Triton X-100 (Wako, Osaka, Japan) for 30 min. After blocking with 5% bovine serum albumin (BSA) (Sigma Aldrich, St. Louis, MO, USA) in PBS (−) for an hour, rabbit γH2AX antibody (Cell Signaling Technology, Danvers, MA, USA) diluted 1:500 in 5% BSA was added to the cells. Then, cells were incubated with the secondary antibody Alexa Fluor 488 anti-rabbit immunoglobulin G (1:1000 dilution; Cell Signaling Technology). After 4′, 6-diamidino-2-phenylindole dihydrochloride (DAPI; Thermo Fisher Scientific) staining, γH2AX formation was measured using a fluorescence microscope (BZ-X800, KEYENCE, Osaka, Japan). More than three independent samples for each condition were used for statistical analysis.

### Statistical analysis

The results are expressed as the average value and SD or represented using box-and-whisker plots with individual data points, showing median and interquartile range (IQR). For the box-and-whisker plots, quartiles were calculated using the inclusive method as specified in the chart settings. Statistical significance was assessed using Student’s T-test, with *P* < 0.05 considered significant. Statistical analysis was performed using Microsoft Excel (Microsoft Corp., Redmond, WA, USA).

## RESULTS

### Reproduced basic beam parameters were validated by measurement

We validated the comparable irradiation setting to previous study [16], nominal physical dose of 7 Gy with nominal dose rate of 100 and 1 Gy/s for uHDR and NDR conditions, respectively. Physical doses were measured using Advanced Markus chamber, showing mean values of 6.99–7.10 Gy with minimal SD within 2% for each irradiation setting. For uHDR condition, short irradiation times within single spill were obtained from the log data derived from monitor chamber. ADR calculated based on acquired dose and irradiation time was 104–106 Gy/s with small SD within 4.0% for uHDR condition. For NDR condition, calculated ADR were 1.0–1.2 Gy/s with relatively large SD due to the variation in number of spills. Nevertheless, the values were sufficiently low to be considered as an NDR. The results of dose, irradiation time and ADR are summarized in Table 1.

**Table 1:** Validation of carbon-ion beam dosimetry

Condition	LETd	Physical dose	Irradiation Time^a^		ADR	Flatness	
	[keV/μm]	[Gy]	[ms]	[s]	[Gy/s]	[%]	
uHDR	16.3	7.02 ± 0.05	67.4 ± 3.1		104 ± 4.2	X	2.6
						Y	1.8
	50.0	7.10 ± 0.09	67.0 ± 2.9		106 ± 3.8	X	3.1
						Y	3.0
NDR	16.3	7.06 ± 0.11		6.8 ± 0.4	1.0 ± 0.06	X	2.3
						Y	1.7
	50.0	6.99 ± 0.05		6.1 ± 1.1	1.2 ± 0.20	X	3.6
						Y	3.1

uHDR: ultra-high dose rate, NDR: normal dose rate, LETd: dose-averaged linear energy transfer, ADR: averaged dose rate

^a^Units differ depending on measurement conditions (s or ms)

**Fig. 2:**
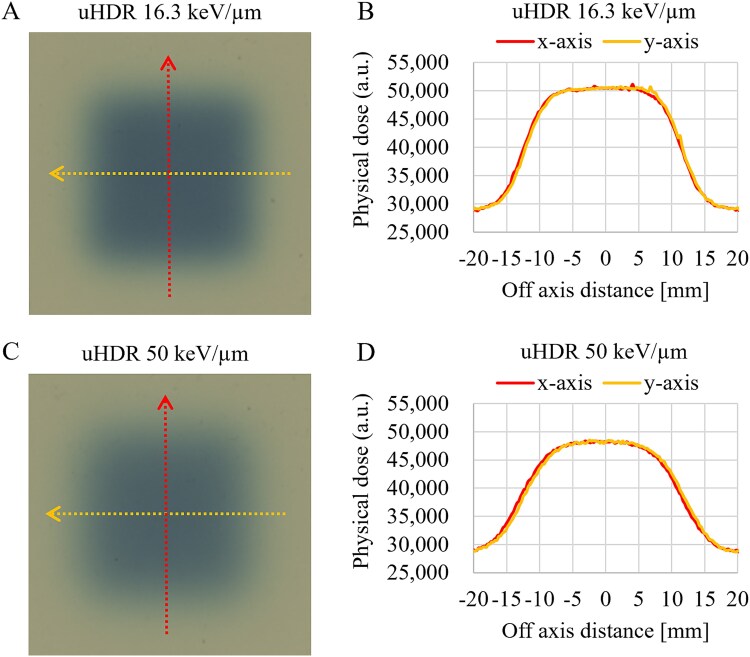
Scanned images and analyzed profiles of Gafchromic films irradiated with 7 Gy of carbon-ion beams under ultra-high dose rate (uHDR) condition with dose-averaged linear energy transfer (LETd) values of 16.3 keV/μm (A, B) and 50.0 keV/μm (C, D). The red and yellow lines indicate x- and y-directions, respectively.

To validate the field flatness for uHDR condition, Gafchromic film was irradiated under plateau and near Bragg-peak region and scanned (Fig. 2A–C). Vertical and lateral dose profiles were extracted from scanned images (Fig. 2B–D). Calculated beam flatness in the range of ±8 mm was 2.6% and 1.8% in the x- and y-directions, respectively, for the plateau region, and 3.1% and 3% for near Bragg-peak region. The field flatness for NDR conditions was also validated in the same manner (Supplementary Fig. 1), showing comparable values to those for uHDR condition. The calculated flatness values for both uHDR and NDR conditions are also included in Table 1.

### The uHDR irradiation of 7 Gy induced the comparable cell-sparing effect with increased surviving fraction and less damage to cell compared to NDR irradiation for normal cell lines under normoxia

**Fig. 3:**
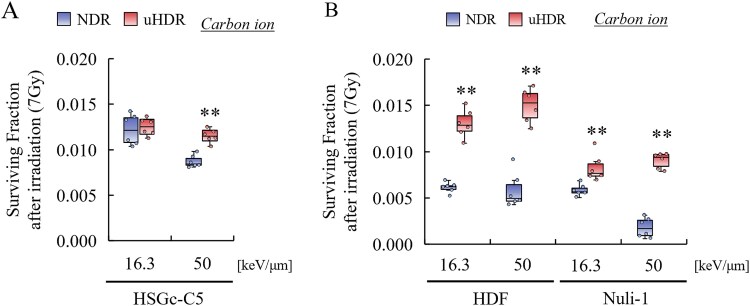
Results of colony formation assay after carbon-ion irradiation with 7 Gy under uHDR or NDR conditions with dose-averaged linear energy transfer (LETd) values of 16.3 or 50 keV/μm. Surviving fractions (SF) of the tumor cell line HSGc-C5(A) and normal cell lines HDF and Nuli-1(B) calculated based on the numbers of seeded cells and formed colonies are shown using box-and-whisker plots with individual data points. For the box-and-whisker plots, quartiles were calculated using the inclusive method as specified in the chart settings. ^**^  *P* < 0.01 vs NDR condition.

To evaluate the surviving fraction after uHDR and NDR irradiation, colony formation assay after carbon-ion irradiation of 7 Gy under normoxia was performed for tumor and normal cell lines. In HSGc-C5 cells, the surviving fraction did not differ significantly between the uHDR and NDR conditions in the plateau region (16.3 keV/μm), but a statistically significant difference was observed under the near Bragg-peak region (50 keV/μm) (Fig. 3A). In contrast, surviving fractions of normal cell lines HDF and Nuli-1 after uHDR irradiation were significantly higher than that for NDR condition regardless of LETd value. Moreover, irradiation with a higher LETd value resulted in a greater difference in the surviving fraction between the uHDR and NDR conditions (Fig. 3B).

**Fig. 4:**
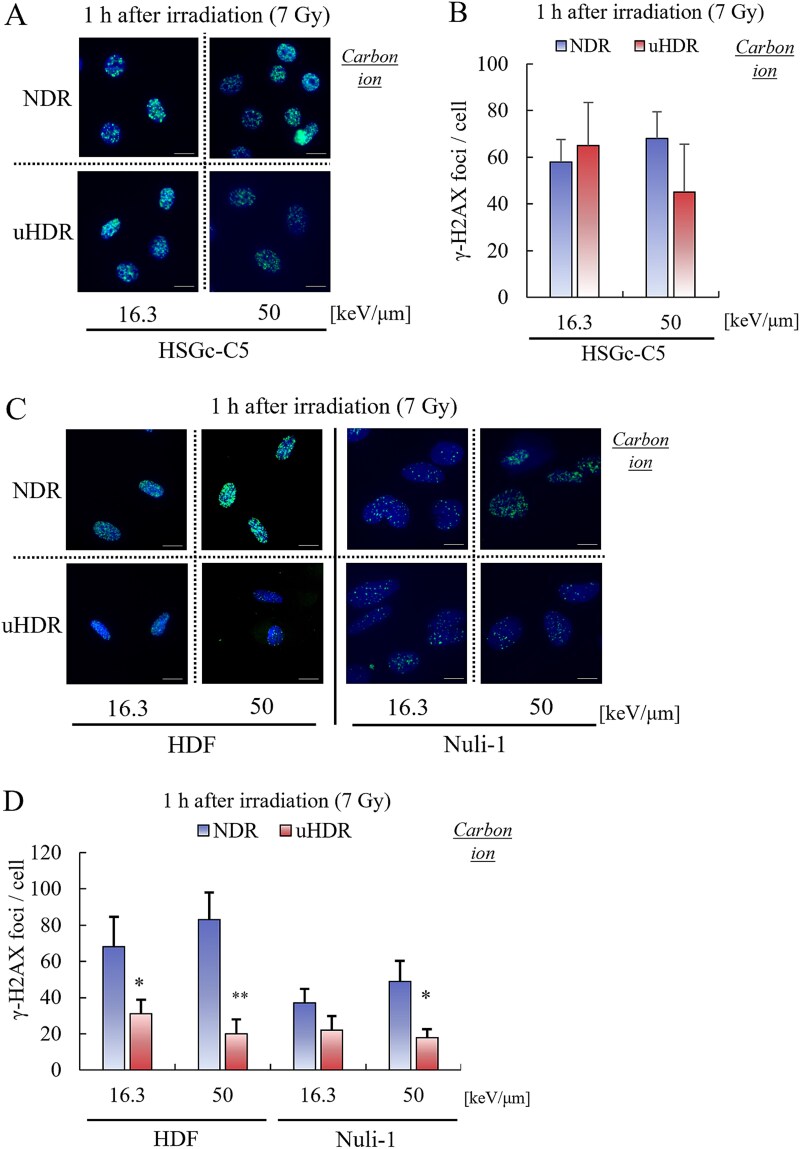
Results of immuno fluorescence staining of γH2AX 1 h after carbon-ion irradiation with 7 Gy under uHDR or NDR conditions with dose-averaged linear energy transfer (LETd) values of 16.3 or 50 keV/μm. Representative immunofluorescence images of γH2AX for the tumor cell line HSGc-C5 (A) and normal cell lines HDF and Nuli-1 (C), with scale bars indicating 10 μm. Numbers of γH2AX foci per cell for the tumor cell line(B) and normal cell lines(D) are shown. Data are presented as mean ± standard deviation. ^*^  *P* < 0.05, ^**^  *P* < 0.01 vs NDR condition.

To evaluate radiation-induced cellular damage, immunofluorescence staining for γH2AX, a marker of DNA double-strand breaks, was performed. For HSGc-C5 cells, there were no significant differences in number of γH2AX foci per cell between uHDR and NDR conditions, although uHDR irradiation with high LETd value (50 keV/μm) tended to be slightly less damage than NDR irradiation (*P* = 0.17) (Fig. 4A–B). In contrast, the number of γH2AX foci per cell for uHDR conditions were lower than that for NDR conditions for HDF and Nuli-1 cells. Additionally, the differences between uHDR and NDR conditions were significant under both the plateau and near Bragg-peak region for HDF, and under only near Bragg-peak region for Nuli-1(Fig. 4C–D). These results for tumor and normal cell lines were consistent with the results of colony formation assay.

Taken together, we successfully observed cell-sparing effects for normal cell lines with increased surviving fraction and less DNA damage to cells after uHDR irradiation under normoxia, while smaller or no cell-sparing effects for tumor cell lines. These results are consistent to the previous research at Osaka-HIMAK [16], except for significant increase in surviving fraction after uHDR irradiation with high LET for tumor cell line under normoxia.

## DISCUSSION

We successfully reproduced and validated the basic irradiation condition including physical dose, ADR and field flatness comparable to previous study at Osaka-HIMAK [16, 43] using the different irradiation system at GHMC. This study is the first approach in the world to create comparable uHDR carbon-ion irradiation conditions for cell experience using different devices. However, there are the other physical beam parameters to consider in the literature of uHDR irradiation and the cell-sparing effect.

In several reports to assess the requirements for the FLASH effect, several approaches to dose rate, besides ADR, were performed. For spot scanning method, several calculation methods for spot and field dose rates, were proposed. Most simple approach is dose rate per spot (DR_spot_), calculated by the total dose of each spot divided by irradiation time of each spot [44]. The other approaches are voxel-based dose rate, such as dose averaged dose rate (DADR) [50] and PBS dose rate [51], considering each cell position and dose contribution to the cell from several spots. In this study comparable field flatness and ADR with same spot alignment as previous studies [16, 43, 44] were adopted, so that DR_spot_ was estimated almost the same, calculated approximately 4900 Gy/s based on ADR and number of scanning spots. Dose contribution from a certain spot depends on not only spot position but dose distribution of single spot. There were small differences in spot size between this study and the previous studies [16, 43, 44] due to machine differences, resulting in possibly small differences in voxel-based dose rate distribution.

**Fig. 5:**
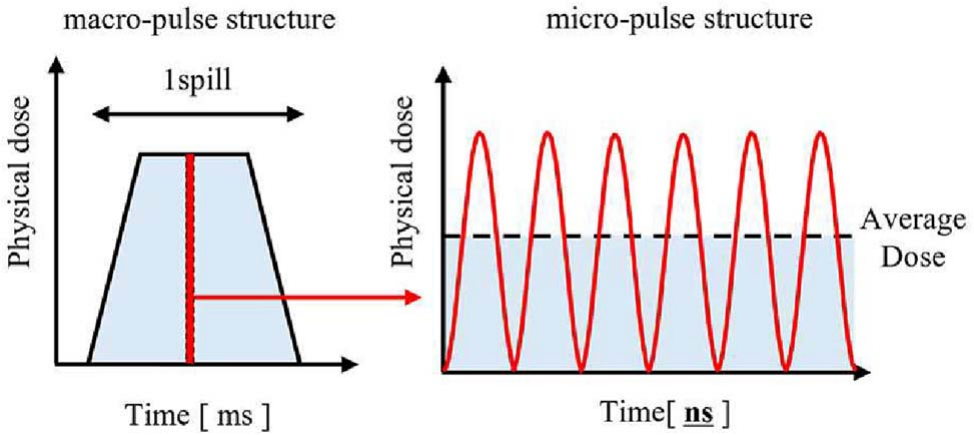
Schematic representation of pulse structures.

For uHDR irradiation of pulsed beam, pulse structures have been reported to be one of the key parameters to induce the FLASH and cell-sparing effect [7, 8, 10, 29] via radical-radical reaction and oxygen consumption in physiochemical and chemical phase of irradiation [2, 28]. The pulse structures are classified into macro-pulse structures in second order and micro-pulse structures in ultra-short time scale, from nanoseconds to femtoseconds as described in Fig. 5. For the beams accelerated by synchrotron, macro-pulse structures can be considered as spills and micro-pulse structures as fluctuations derived from Radiofrequency (RF) acceleration pulse. For macro-pulse structures, each irradiation for uHDR conditions was delivered in one spill in this study. Thus, based on the framework proposed by N. Esplen *et al.* [29], the pulse width, DPP, and $ \dot{\mathrm D}$_p_ for macro-pulse structure of each spot were calculated as approximately 1.4 ms, 7 Gy, and 5100 Gy/s, respectively, according to Table 1 and the number of scanning spots. These values were consistent with those reported in previous studies [16, 43, 44]. For micro-pulse structures, PRF could be estimated 6.13 MHz based on RF pulse frequency and DPP were calculated about 8.4 × 10^−4^ Gy/pulse based on physical dose, PRF and number of scanning spots in this study. These values were comparable to the previous research for low LETd irradiation (6.10 MHz and 8.54 × 10^−4^ Gy/pulse) [16, 44]. Pulse width and $ \dot{\mathrm D}$_p_ could not be directly measured because of the extremely short timescale involved. Nevertheless, maximum pulse width could be estimated 163 ns under the assumption of no interval between micro-pulses, resulting in $ \dot{\mathrm D}$_p_ > 5.1 × 10^3^ Gy/s. Based on these estimations, micro-pulse structure in this study is considered to be not substantially different from previous studies at least for low LETd irradiation [16, 43, 44].

We successfully observed cell-sparing effects after uHDR carbon-ion irradiation under normoxia for normal cells, while no or smaller cell-sparing effects for tumor cells. In the results of both colony formation assay and immunofluorescence staining of γH2AX, irradiation with higher LETd induce more difference between uHDR and NDR conditions. These results are comparable to the previous study of Minami *et al.* [16]. In addition, under NDR condition, higher LETd irradiation resulted in higher biological effectiveness, i.e. higher relative biological effectiveness (RBE) for carbon-ion irradiation in the LET range used in this study [36–38]. Though we did not assess the RBE in this study, the RBE at 10% survival (RBE_10_) for the cell lines used in this study based on *in vitro* clonogenic survival has been reported in only a few articles [39–42]. Based on the data reported by Kagawa *et al.* [39], RBE_10_ values for HSG at LETd of 16.3 and 50 keV/μm can be estimated by interpolation as 1.27 and 1.83, respectively. The physical dose of 7 Gy therefore corresponds to biological equivalent dose (BED) of approximately 8.9 and 12.8 Gy-RBE based on these RBE_10_ values. In addition, HDF may have RBE values similar to those of HSG, while NuLi-1 may exhibit higher RBE values according to the report of Yagi *et al.* [42]. Moreover RBE-based dose rate is higher in higher LET irradiation based on these estimations. In this study, higher BED under near Bragg-peak region with higher RBE-based dose rate might have contributed to the significant cell-sparing effect. In other words, physical dose threshold for the cell-sparing effect might be lower for higher LETd irradiation delivered at the same physical dose rate. In consistent with these results, for proton beam with lower LET and smaller RBE of 1.1 generally in the previous studies, no sparing effects were detected below 15 Gy irradiation [20–22] and much higher dose exceeding 15 Gy was required to induce the cell-sparing effect under normoxia [15, 22]. Furthermore, for low LET electron beams, a dose higher than 10 Gy was required to observe cell sparing effect under normoxia [5, 52]. However, because RBE incorporates both direct and indirect actions of radiation, it does not fully account for the dose threshold. According to the radical recombination hypothesis, uHDR irradiation with higher LET produces a greater radical density along particle tracks, promoting radical recombination and consequently reduces ROS-mediated damage via indirect action [33]. Nonetheless, even for high-LET radiation, such as carbon ions, indirect action still contributes to cell viability to some extent in addition to direct action [53]. Taken together, these considerations indicate that RBE alone cannot fully explain the dose threshold, highlighting the importance of considering the dose threshold individually for each LET. Although differences in RBE and radical recombination may be possible contributors to the LET dependence in the cell sparing effect, further investigation is needed to elucidate the underlying mechanism of cell sparing effect for high LET irradiation under normoxia.

For further investigation into requirements for the cell-sparing effect in carbon-ion irradiation, irradiation conditions, as mentioned above, biological experiments and whether the cell-sparing effect observed or not are summarized in Table 2 for this study and previous reports conducting *in vitro* experiments after uHDR carbon-ion irradiation [14, 16, 23].

**Table 2:** Comparison of irradiation conditions and biological assessments for *in vitro* studies of uHDR carbon-ion irradiation

Publication	This Study	Minami *et al.* [16]	Tashiro *et al.* [23]	Tinganelli *et al.* [14]
Institution	Gunma University Heavy Ion Medical Center (GHMC)	Osaka Heavy Ion Therapy Center (Osaka-HIMAK)	Gunma University Heavy Ion Medical Center (GHMC)	Heidelberg Ion-Beam Therapy Center (HIT)
Carbon-ion irradiation system	MELTHEA (Synchrotron, Hitachi, Ltd.)	HyBEAT (Synchrotron, Hitachi, Ltd.)	MELTHEA (Synchrotron, Hitachi, Ltd.)	HIT accelerator system (Synchrotron)
Beam Energy [MeV/u] (plateau / near Bragg-peak region)	290	208.3 / 100	290	280
LETd [keV/μm] (plateau / near Bragg-peak region)	16.3 / 50.0	19.0 / 50.0	13 / 50	13 / N.A.
Maximum physical dose [Gy]	7.0	6.5	3	7.5
Averaged dose rate [Gy/s]	100 / 1.0	100 / 1.0	96–195 / 7.7–12	70 / 0.6
Spot alignment	7 × 7 spots 3 mm spacing	7 × 7 spots 3 mm spacing	Single spot	4 × 4 spots 3 mm spacing
Spot size [mm]	2.4 (in air / SD)	3.0 (in air / SD)	2.4 (in air / SD)	5 (FWHM)
RF pulse frequency [MHz] (plateau / near Bragg-peak region)	6.13	6.10 / N.A.	6.13	N.A.
Cell lines (tumor / normal cells)	HSGc-C5 / HDF, Nuli-1	HSGc-C5 / HDF, Nuli-1	HSGc-C5 / HFL1	N.A./ CHO-K1
Cell culture plate	24-well plate	24-well plate	96-well plate	Gas-permeable foil with 8 mm phi ring
Oxygen concentration	Normoxia (21%)	Normoxia (21%) Hypoxia (4%)	Normoxia (21%)	Normoxia (21%) Hypoxia (4%,0.5%), Anoxia (0%)
Biological experiments to assess cell sparing effect	Colony formation assay Immunofluorescent staining of γ-H2AX	Colony formation assay Immunofluorescent staining of γ-H2AX	Clonogenic assay Cell proliferation assay SA-β-gal staining	Clonogenic assay
Cell sparing effect under normoxia (tumor / normal cells)	Partially Yes^a^ / Yes	No / Yes	No / No	N.A. / No
Cell sparing effect under hypoxia (tumor / normal cells)	N.A.	Partially Yes^a^ / Yes	No / No	N.A. / Yes

LETd: dose averaged LET, HFL1: human lung fibroblast， CHO-K1: Chinese hamster ovary cell, SA-β-gal: senescence associated β-galactosidase, N.S.: not assessed

^a^Cell sparing effect was observed under near Bragg-peak region, but not under plateau region.

For tumor cells, small but significant difference in surviving fraction after irradiation with high LETd values (50 keV/μm) was observed in this study, which was not demonstrated under normoxia in the other reports in Table 2, but under hypoxia in the previous report of Minami *et al.* [16]. The cell-sparing effect for tumor cells under normoxia has been reported in other reports using other types of beams [5, 15, 17]. Additionally, the cell-sparing effect was induced with lower dose under hypoxia than under normoxia for normal cell lines [16]. These findings in previous studies can be explained based on oxygen depletion hypothesis which has been proposed since the early stages of uHDR irradiation research. Transient oxygen consumption and the resulting decrease in oxygen levels induced by uHDR irradiation increase the surviving fraction to greater extent under hypoxia than under normoxia [2, 12, 13, 31, 32]. In this study, a slightly higher physical dose of 7 Gy was adopted, compared with 6.5 Gy in the previous study of Minami *et al.* [16]. This result indicates that the dose threshold for HSGc-C5 under normoxia might be between 6.5 and 7.0 Gy with LETd of 50 keV/μm, higher than that under hypoxia, as is the case for normal cell lines. However, it has been reported that the cell-sparing effect in tumor cells varies depending on tumor type and oxygen concentration [54], warranting further investigation using other types of tumor cells and different oxygen conditions.

For normal cells, sparing effect was not observed in a previous study of Tashiro *et al.* using the same machine at GHMC with physical dose of 3 Gy and comparable or higher ADR [23]. It is suggested, in addition to high dose rate, higher physical dose over 5 to 7 Gy are needed to induce the cell-sparing effect under normoxia than that under hypoxia as reported in the previous research [16]. However, Tinganelli *et al.* reported the cell-sparing effect after higher physical dose irradiation (7.5 Gy) was not observed under normoxia but was observed under hypoxia [14]. In this article, a smaller number of cells due to the limitation of field size were irradiated under plateau region. It suggests that higher LETd value, namely higher BED for NDR irradiation, and a sufficient number of cells might be needed to induce and observe the cell-sparing effect under normoxia than under hypoxia. Moreover, the differences in normal cell lines might affect the results, as suggested by the small difference in the surviving fraction and DNA damage observed between the two cell lines in this study.

As described in Table 2, in addition to physical dose, there was small difference in beam energy, LETd value of plateau region, voxel-based dose rate via spot size, and micro-pulse structures, which depends on the irradiation system, compared to the previous reports of Minami *et al.* [16]. However, the comparable cell-sparing effects were observed. It is suggested that these small differences might have slight effect on the cell-sparing effect compared to the other beam parameters. Our findings of the requirements, such as dose, dose rate, and LETd value, for the cell-sparing effect under normoxia for the cell lines used in the previous study were validated to be independent from irradiation devices.

For multi-institutional pre-clinical studies and clinical trials of uHDR irradiation, validation of dosimetric parameters has been reported to be necessary to ensure the reliability of biological and clinical outcomes [55–57]. Based on the results of this study, slight differences in physical dose and LET were found to affect the biological results for carbon-ion uHDR irradiation; therefore, at least the parameters listed in Table 2 are encouraged to be validated. Moreover, further exploration of biological mechanisms and clinically applicable physical settings, such as widening the irradiation field laterally and in depth including the creation of Spread-Out Bragg-peak (SOBP), is needed for the clinical translation of carbon-ion uHDR irradiation. This study suggests that comparable carbon-ion uHDR irradiation with validated beam parameters is expected to induce equivalent cell-sparing effects independently of irradiation machines at least for the cell lines used in this study. These insights may expand the use of the limited number of available machines worldwide, promoting FLASH research and ultimately leading to clinical trials toward the implementation of instantaneous volumetric irradiation (IVI), after many challenges such as limitations in irradiation volume are resolved in the future.

In conclusion, we achieved comparable beam parameters for uHDR carbon-ion irradiation using a different machine and demonstrated cell-sparing effects consistent with previous studies [16, 43]. This study is the first in the world to indicate that the cell-sparing effect is not dependent on irradiation devices but rather on beam parameters and irradiation conditions, which may accelerate the research and promote the clinical implementation of uHDR carbon-ion irradiation.

## Supplementary Material

Revised_Supplementary_Figure1_no_highlight_rrag039

## PRESENTATION AT A CONFERENCE

This study was presented at 5th Flash Radiotherapy and Particle Therapy Conference (FRPT 2025) and 38th Annual Meeting of Japanese Society for Radiation Oncology (JASTRO).
