# Supplementary material for: For ultra-high dose rate carbon-ion irradiation, comparable beam parameters induce the equivalent cell sparing (FLASH) effect
Source: J Radiat Res. 2026 Jun 3;67(4):487–97. doi: 10.1093/jrr/rrag039 (PMC13400559; doi:10.1093/jrr/rrag039)
Supplement: Revised_Supplementary_Figure1_no_highlight_rrag039 [file revised_supplementary_figure1_no_highlight_rrag039.docx]

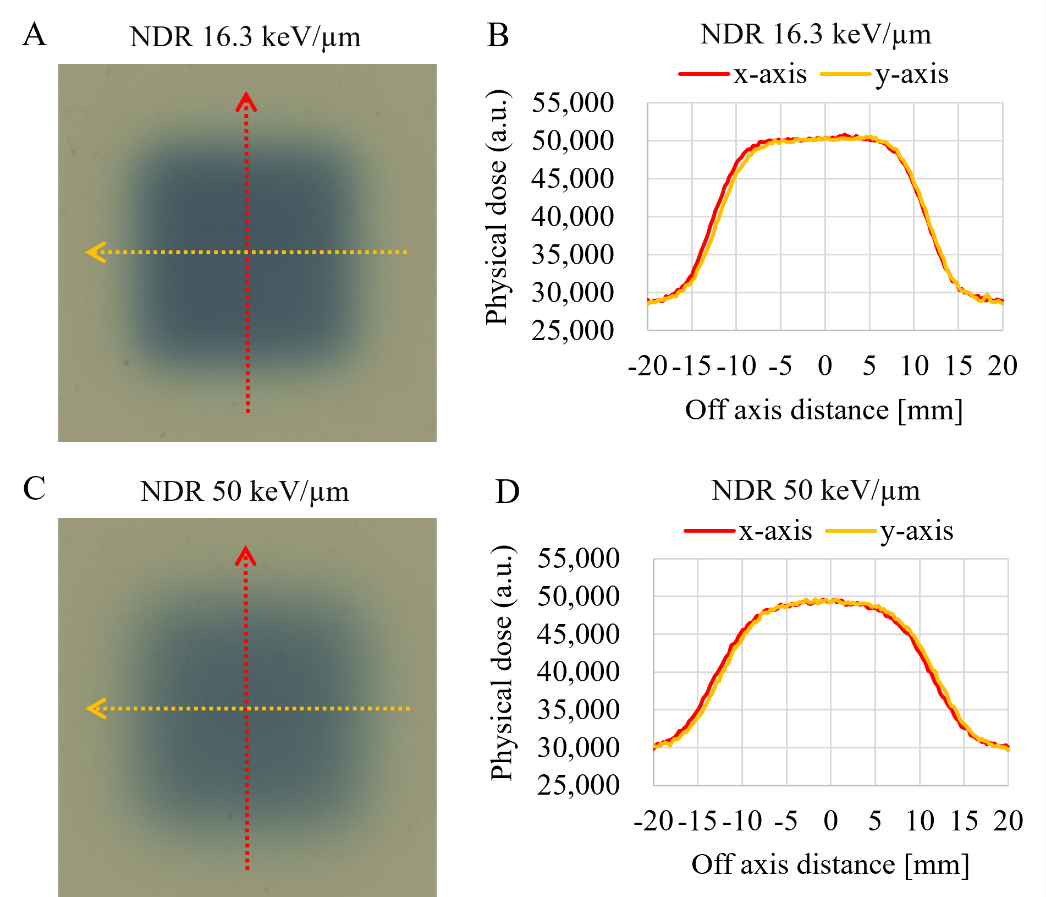


Supplementary Figure 1. Scanned images and analyzed profiles of Gafchromic films irradiated with 7 Gy of carbon-ion beams under normal dose rate (NDR) condition with dose-averaged linear energy transfer (LETd) values of 16.3 keV/μm (A, B) and 50.0 keV/μm (C, D). The red and yellow lines indicate x- and y-directions, respectively.

Alt Text: Scanned images and corresponding dose profiles of Gafchromic films after 7 Gy carbon-ion irradiation under normal dose rate (NDR) conditions with each dose-averaged linear energy transfer (LETd) value. The profiles along the x- and y-directions were flat within ±8 mm.
